# Impact of a dedicated center for atrial fibrillation on resource utilization and costs

**DOI:** 10.1002/clc.23974

**Published:** 2023-01-20

**Authors:** Ankit Medhekar, Suresh Mulukutla, Whitney Adams, Amanda Kristofik, Erica Byers, Floyd Thoma, Konstantinos Aronis, William Barrington, Raveen Bazaz, Aditya Bhonsale, Nathan Anthony Mark Estes, Krishna Kancharla, Andrew Voigt, Norman C. Wang, Samir Saba, Sandeep K. Jain

**Affiliations:** ^1^ Department of Medicine, Section of Cardiology Baylor College of Medicine Houston Texas USA; ^2^ Heart and Vascular Institute at the University of Pittsburgh Medical Center Pittsburgh Pennsylvania USA

**Keywords:** atrial fibrillation, cost, quality

## Abstract

**Background:**

Atrial fibrillation (AF) affects millions of Americans each year and can lead to high levels of resource utilization through emergency department (ED) visits and inpatient stays.

**Hypothesis:**

We hypothesized that referral of patients to a dedicated Center for AF from the ED would reduce costs of care.

**Methods:**

The University of Pittsburgh Center for AF serves as a rapid referral center for patients with AF to avoid unnecessary inpatient admissions and provide specialized care. Patients that presented to the ED with AF and met prespecified criteria were directed to rapid outpatient follow‐up instead of inpatient admission. The primary outcome of interest was 30‐day total costs. Secondary outcomes included outpatient costs, inpatient costs, 90‐day costs, and inpatient stay characteristics.

**Results:**

We identified 96 patients (median age 65, 38% women) referred to the center for AF for a new diagnosis of AF between October 2017 and December 2019 and matched 96 control patients. After 30 days of follow‐up, patients referred to the center for AF had a lower average cost ($619 vs. $1252, *p* < 0.001) compared to controls, driven by lower costs of ED care tempered by slightly higher outpatient costs. Thirty‐day admissions and lengths of stay were also lower. These differences were persistent at 90 days.

**Conclusion:**

Directing patients with AF that present to the ED to follow‐up at a dedicated Center for AF significantly reduced overall costs, while reducing subsequent inpatient admissions and total lengths of stay in the hospital.

## 1 INTRODUCTION

Atrial fibrillation (AF) is the most common arrythmia in the United States, affecting 1.1% of the US population.^1^ The presence of AF can be a harbinger of a variety of adverse outcomes including a fivefold greater increased risk of stroke, an increased risk of death and heart failure, and a greater likelihood of hospital admission.^2^ In fact, there are almost 500 000 inpatient admissions for AF each year, contributing to substantial costs to the healthcare system.^3^

National analyses of cost for AF have estimated that the total cost of AF in the United States is $6−$26 billion dollars each year.^4^, ^5^ It is believed that these costs may be driven by complications of AF, such as stroke and inpatient hospitalization.^6^ Recognizing the substantial burden of AF on patients in the United States, the Heart Rhythm Society (HRS) has recommended that clinicians and healthcare facilities transition to use of AF centers. The goal of such a center would be to “improve outcomes by providing a better patient experience and a delivering high‐quality, guideline recommended, state‐of‐the‐art care.”^7^ The HRS strongly supports utilization of centers like these to improve patient outcomes. However, while the HRS provides substantial evidence for improved outcomes with AF care clinics, there exists little evidence regarding the impact of a center for AF on cost. We sought to understand changes in resource utilization and healthcare expenditure associated with use of a dedicated Center for Atrial Fibrillation.

## 2 METHODS

**Figure 1:**
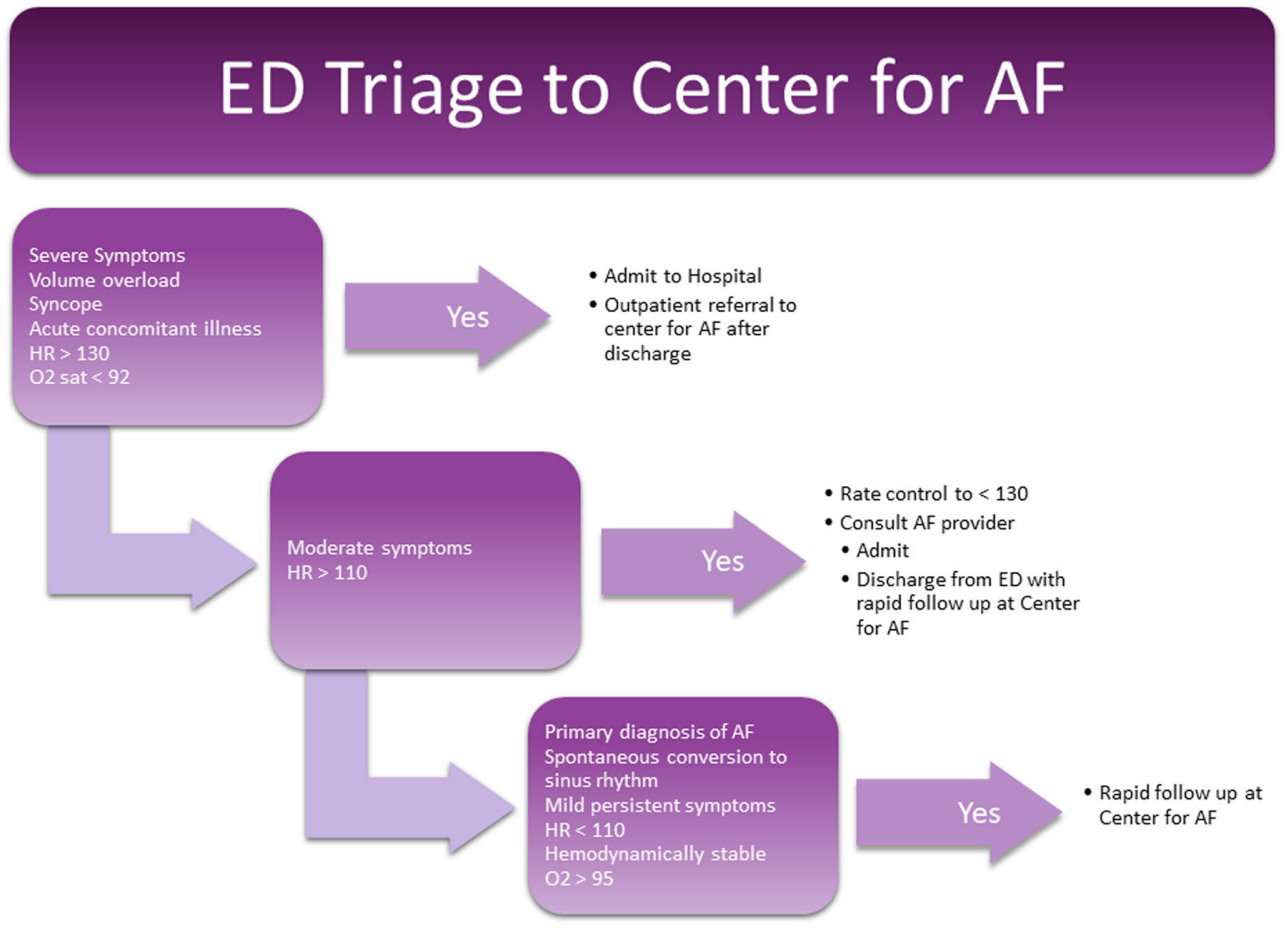
Center for AF ED triage algorithm for patients presenting with AF as their primary problem. AF, atrial fibrillation; ED, emergency department.

In 2017, we created a specific pathway for management of AF patients presenting to the emergency department (ED) through a dedicated Center for AF in our institution. Our goals included diminishing unnecessary AF attributable hospital encounters, providing meaningful education to patients, and providing expertise in AF treatment options. We provided a 24/7 consultative service that offered an initial educational and “triage” visit within 24 h with an advanced practice provider supervised by an electrophysiologist. Patients that received care at the Center for AF were offered expedited options for rhythm control, devices, ablation, left atrial appendage occlusion devices, and antiarrhythmic medications as appropriate. Additionally, patients receive specialized and detailed education on their disease process, anticoagulation, lifestyle factors, and procedural options for controlling AF. We sought to use the Center as a mechanism to provide rapid outpatient follow‐up for stable patients diagnosed with AF that presented to the ED with a first episode of AF. Patients were triaged using the algorithm shown in Figure 1. Our strategy was adapted from an approach used by clinicians at the University of North Carolina.^8^

We identified patients who presented to an ED in 1 of 17 hospitals within our network with new AF who met prespecified criteria for potential outpatient management based on heart rate and clinical stability and were referred to our Center for AF from hospitals within our system. Contemporaneous propensity matched controls were identified among patients who were not referred to the Center for AF. Patients were matched by age, BMI, and medical history of hypertension, hyperlipidemia, stroke, CKD, congestive heart failure (CHF), COPD, CAD, cirrhosis, active cancer, and diabetes. Patient cost data was obtained for each patient by encounter.

Our primary outcome measure was total 30‐day costs after the index presentation. Cost data within the UPMC health system was obtained subcategorized as total expenditures for all encounters, total expenditures for outpatient encounters, and total expenditures for ED encounters. We also identified 30‐day costs (total, outpatient, and ED), cardiac admissions, AF admissions, and total length of stay characteristics as outcome measures of interest.

A bivariate analysis using Pearson's *χ* ^2^ test, Student *t*‐test, and Mann−Whitney *U* test was performed as appropriate to the data to determine differences in baseline characteristics. Important differences in baseline characteristics were referred to subsequent sensitivity analysis. Quartiles of cost were determined as was the interquartile range. Costs greater than 1.5x the interquartile range above the third quartile (i.e., costs > Q3 + 1.5 × IQR) were labeled as outliers and were removed. Median costs were then compared using the Wilcoxon rank sum.

Admissions were manually reviewed, and the type of admission was determined by individual review of primary diagnosis code. Admissions labeled AF (ICD‐10 I48.0, I48.1, I48.19, I48.91) were also labeled as cardiac admissions. Non‐AF cardiac admissions were individually reviewed and included diagnoses in the ICD‐10 Ixx subcategory and “chest pain.” Mean cardiac and AF admissions were calculated and compared between groups using the Student's *t*‐test. Median length of stay was determined in each group and lengths of stay were compared using Wilcoxon rank sum. This study was approved by the University of Pittsburgh Institutional Review Board.

## 3 RESULTS

Baseline characteristics of the population are as shown in Table 1. In summary, 192 patients with average age 65 were identified, of which 44% were women.

**Table 1:** Baseline characteristics

Variable	AF center (*n* = 96)	Control (*n* = 96)	*p* Value
Age (median, IQR)	65.00 [55.00−71.25]	62.00 [53.00−74.25]	0.951
Follow‐up days (median, IQR)	699.50 [397.75−847.25]	650.00 [450.50−863.25]	0.824
Female (%)	38 (39.6)	46 (47.9)	0.309
Caucasian (%)	88 (91.7)	93 (96.9)	0.214
BMI (median, IQR)	29.34 [25.95−34.80]	30.84 [25.90−34.99]	0.904
Hypertension	55 (57.3)	49 (51.0)	0.469
Diabetes	16 (16.7)	14 (14.6)	0.842
CHF	5 (5.2)	15 (15.6)	0.033
Stroke	4 (4.2)	5 (5.2)	1
Cirrhosis	2 (2.1)	0 (0.0)	0.497
COPD	10 (10.4)	13 (13.5)	0.657
CAD	6 (6.2)	9 (9.4)	0.591
HLD	53 (55.2)	50 (52.1)	0.772
CKD	5 (5.2)	3 (3.1)	0.721
Active cancer	13 (13.5)	11 (11.5)	0.827

Abbreviations: AF, atrial fibrillation; CHF, congestive heart failure.

**Figure (2A−C):**
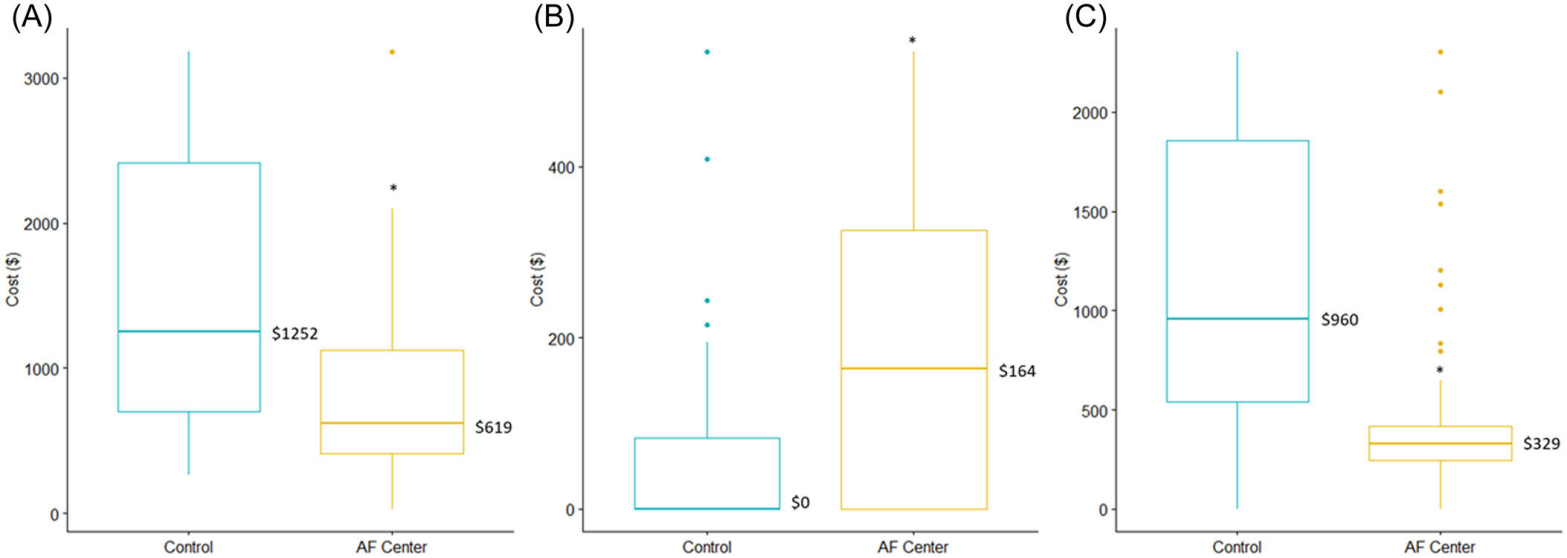
Thirty‐day. (A) Total, (B) outpatient, and (C) emergency department costs between the groups. *p < 0.001.

**Figure (3A−C):**
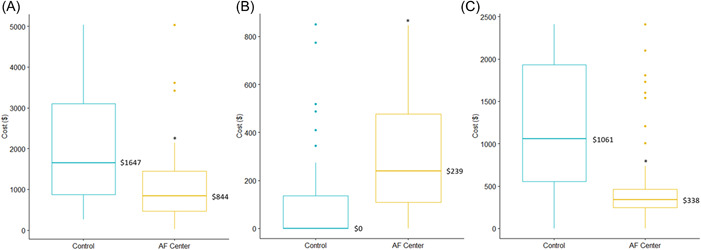
Ninety day. (A) Total, (B) outpatient, and (C) emergency department costs between the groups. *p < 0.001.

At 30 days, total costs and ED costs were lower in patients referred to the Center for AF compared to controls, whereas outpatient costs were slightly higher in patients referred to the Center for AF than controls (Figure 2A−C). These differences persisted in the 90 day analysis (Figure 3A−C). At 30 days, patients referred to the center for AF had fewer cardiac (6 vs. 22, *p* < 0.001) and AF (4 vs. 19, *p* < 0.001) admissions compared to control patients. They also had shorter lengths of stay (0.17 vs. 0.83, *p* < 0.001) (Table 2). These findings were persistent after 90 days (Table 3). CHF patients were found to be slightly more common in the control group. Mortality outcomes were not different between the groups. A sensitivity analysis was performed excluding all patients with CHF and results were similar to those above.

**Table 2:** Average admissions per patient by type and total length of stay for any admission 30 days after index visit

30 day outcomes			
	AF center	Control	*p* Value
Cardiac admissions	6	22	0.001
AF admissions	4	19	0.001
Length of stay	0.167	0.83	0.001

Abbreviation: AF, atrial fibrillation.

**Table 3:** Average admissions per patient by type and total length of stay for any admission 90 days after index visit

90 day outcomes			
	AF center	Control	*p* Value
Cardiac admissions	10	33	0.001
AF admissions	8	26	0.001
Length of stay	0.167	1.16	0.001

Abbreviation: AF, atrial fibrillation.

## 4 DISCUSSION

In this retrospective, single institution study of a dedicated center for AF, we demonstrated that patients referred to the Center had lower costs and fewer inpatient stays. Reductions in cost were principally driven by reductions in ED expenditure. Patients referred to the Center for AF had slightly higher outpatient costs, likely reflecting that these patients utilized outpatient care for their AF rather than ED care. Patients in the Center for AF also had notably lower cardiac and AF related admissions with overall lower lengths of stay.

AF is an important contributor to healthcare costs in the United States. The biggest contributor of this cost appears to be inpatient hospitalizations.^9^ Our study demonstrated that patients referred to the Center for AF had lower inpatient admissions and shorter lengths of stay—meaning less utilization of inpatient resources.

While there do not appear to be other studies that specifically focused on cost reductions through a dedicated center for AF, others internationally have demonstrated reductions in cost through a directed approach in treating AF. In Italy, Pastori and colleagues analyzed the effects of using an “Atrial Fibrillation Better Care (ABC)” pathway in cost reduction.^10^, ^11^ They found that in patients that had optimal management under the ABC pathway had a cost savings of $3137 (USD) per patient over a median follow‐up of 36.9 months. Notably, this was an unadjusted, descriptive analysis and did not account for confounders that resulted in facets impairing optimal management. Additionally, unlike our study which was able to use direct costs borne by the hospital, Pastori et al. were forced to use reimbursement estimates driven by diagnosis related groups. In the same vein as Pastori, Pathak et al.,^12^ in Australia created a risk factor management clinic for AF and performed a cost effectiveness analysis to compare to patients that refused the clinic. Through their cost estimations, they determined a cost savings of $62 653 (AUD) per QALY gained. This estimation is somewhat difficult to compare to ours given the difference in cost estimation but provides evidence that use of a dedicated center for AF may both be associated with cost reduction as well as improved long‐term outcomes.

Others have also shown that use of a center for AF may be impactful in improving outcomes, which can be a key driver in improving costs.^13^ Stroke is one of the most feared complications of AF and can be a major driver of cost.^10^ 1 in 10 deaths related to AF are due to stroke.^14^ In our program as well as similar programs, one of the most important goals is to assure adequate anticoagulation. The cost benefits of anticoagulation in AF are well described and one study suggests that these benefits may in fact be currently underestimated.^15^ An international study demonstrated that an educational intervention for AF, a characteristic of our center for AF, increased rates of anticoagulation and decreased stroke.^16^ Other studies have also demonstrated that the use of coordinated systems of care in AFs may have far reaching benefits including reduced all‐cause and cardiovascular mortality, reduced cardiovascular hospitalizations, and ED visits.^17^, ^18^, ^19^, ^20^

We note that our study does have limitations. First it is a retrospective cohort study of a single hospital system. It is possible that we may have biases that are inherent to this type of analysis, such as the potential for unmeasured confounding variables and referral bias. As we are only studying our system, we are unable to obtain costs of care that patients experienced outside of our system that may impact the data. Additionally, as we had not implemented the center for AF at each institution in our system, there is a possibility of geographic referral biases. This is balanced by the significant advantage of the ability to have contemporaneous control groups that rely on this geographic differential. Our control groups were also spread over nine hospitals, which further limits this possibility. Second, we rely on appropriate coding of diagnoses and treatments for AF. Inaccuracies or inconsistencies in reporting would affect the quality of our data. Finally, we acknowledge that as our center for AF is relatively young, our study has a smaller *n* than other similar studies. Nevertheless, we were able to show a statistically and clinically significant reduction in costs as well as admissions.

## 5 CONCLUSION

As our healthcare system transitions into payment models that are driven by quality and that aim to reduce costs, utilization of dedicated centers like our Center for AF will be invaluable in providing high‐quality, cost‐effective care. Our data suggests that patients that presented to our institution that were referred to our Center for AF not only had lower costs but spent less time in the hospital. Further research as our center and others grow will be helpful in better quantifying the magnitude of cost reduction, demonstrating improvement in outcomes, and suggesting avenues for further cost savings.

## Data Availability

The data that support the findings of this study are available from the corresponding author upon reasonable request.
